# Bridging the bioinformatics gap: tool selection for decentralized AMR genomic surveillance in Africa

**DOI:** 10.3389/fpubh.2026.1756324

**Published:** 2026-04-09

**Authors:** Niamh Lacy-Roberts, Christa Twyford Gibson, Pernille Nilsson, Colette J. Weese, Christina Odgaard, Natasia Rebekka Thornval, Rene S. Hendriksen

**Affiliations:** Research Group for Global Capacity Building, National Food Institute, Technical University of Denmark, Kgs. Lyngby, Denmark

**Keywords:** Africa, antimicrobial resistance, bioinformatic pipelines, genomic surveillance, low-and middle-income countries

## Abstract

Antimicrobial resistance (AMR) poses a significant threat to public health, particularly in low- and middle-income countries where centralized genomic surveillance infrastructure is limited. To support decentralized AMR surveillance in Africa, the SeqAfrica project evaluated bioinformatics tools suitable for Oxford Nanopore Technologies sequencing data. Through two surveys and an expert elicitation workshop, the project assessed over 80 tools, focusing on usability, cost, accessibility, and AMR relevance. Graphical user interface (GUI) based tools were shortlisted for non-bioinformatician use. Terra.bio and EPI2ME emerged as leading candidates due to their user-friendly interfaces and integrated workflows. EPI2ME, which is an Oxford Nanopore Technologies tool, was favored for its offline capability, ease of use, and compatibility with local infrastructure, while Terra.bio offered strong interoperability and cloud-based scalability. The findings underscore the need for standardized, interoperable pipelines, and sustained technical support to ensure effective genomic surveillance in resource-limited settings. SeqAfrica has initiated pilot implementation and collaborative development of EPI2ME-based workflows to enhance AMR monitoring capacity across the African continent.

## 1 Introduction

The increasing resistance of bacteria, fungi, and viruses to antimicrobials critical to public health and agricultural systems disproportionately exacerbates disease burden, mortality rates, and food insecurity in low- and middle-income countries (LMICs) (1). This impact is already evident in Sub-Saharan Africa, which is disproportionately affected by antimicrobial resistance (AMR) due to factors including poor regulation, ineffective or poor supply of antibiotics, and high burden of infectious diseases (2). Murray et al. (3) found a high level of AMR burden for several bacterial pathogens associated with high mortality rates in Africa. Genomic surveillance using whole genome sequencing (WGS) technologies can play a role in fighting the spread of AMR. WGS can provide a comprehensive view of the genetic make-up of pathogens, allowing scientists and policymakers to understand resistance mechanisms, track the evolution and spread of pathogens and AMR genes, and detect emerging threats (4).

To leverage the power of WGS in the fight against AMR, the UK International Development Fleming Fund SeqAfrica project has been working to build sequencing capacity across Sub-Saharan Africa. The project provides sequencing services, technical assistance, training, and simulated exercises to scientific staff across Sub-Saharan Africa, supporting skills development for conducting outbreak investigations and tracking of resistance genes across human, animal, and environmental sectors under a One Health approach.

As part of the SeqAfrica project’s efforts to democratize sequencing on the African continent, a pilot project was introduced in 2024 at two laboratories in South Africa. The aim of the pilot was to determine the feasibility of de-centralizing WGS surveillance of AMR in Africa. One common centralized surveillance method requires that isolates of concern are shipped to a National Reference Laboratory for sequencing, which can cause delays and added expense. By implementing Oxford Nanopore Technologies (ONT) platforms in smaller ancillary laboratories, SeqAfrica aims to provide empirical evidence supporting the feasibility of decentralized AMR surveillance at the local level. ONT’s MinION sequencing platform is a palm sized device that can be more affordable than traditional sequencing technologies [MinION (RRID: SCR_017985)] for smaller laboratories due to low start-up costs and fewer set-up requirements. While the laboratory procedures for using the MinION are relatively straight forward, identifying an analysis protocol that could be used by researchers with limited bioinformatics skills can pose a challenge. ONT generates long reads, which are advantageous for resolving complex genomic regions, but which are also associated with higher error rates compared to short-read sequencing platforms like Illumina. These characteristics necessitate the use of specialized analytical tools designed to handle long-read sequencing data and perform robust error correction. Furthermore, most existing bioinformatic tools are designed to work with short read data and need to be adapted for analysis of long-reads. ONT produces data in specific formats such as FASTQ (base-called reads) and POD5 (raw signal data), which further influences tool compatibility and workflow design.

## 2 Methods

To address the challenges described above, we initiated a search for a bioinformatics analytical platform capable of performing comprehensive quality control (QC), genome assembly, species identification, multilocus sequence typing (MLST), AMR determinant (acquired resistance genes and resistance-associated point mutations) detection, and phylogenetic analysis (5). Selection criteria for the tool were identified. In applying these criteria, we evaluated each tool according to how many of the predefined criteria it could meet. Our aim was to identify a tool that satisfied the maximum number of criteria rather than to exclude tools that failed to meet a specific minimum. No benchmarking or accuracy comparisons were conducted.

- Cost: The tool should be low or no-cost to allow for introduction and sustainability in LMICs.
- Ease of use: The tool should be easy-to-use and intuitive [e.g., a graphical user interface (GUI)] for a non-bioinformatician. It should also be easy to install and/or configure.
- Resource requirements: The tool should be able to run on a standard-to-high-end laptop or desktop without the need for a powerful computer [e.g., high random access memory (RAM)].
- Accessibility: Because the tool will be used in LMIC settings with inconsistent and often slow internet coverage, the tool should ideally operate offline or on a local server and use very low bandwidth.
- Interoperability: The tool should produce outputs that can be used by other systems and should be able to plug into other workflows.
- AMR Relevance: The tool should be able to complete an analysis pipeline that detects AMR determinants, ideally within a ready-made workflow and not a series of tools that the user would need to string together.

With these criteria in mind, we conducted an initial semi-structured survey that was sent to 15 SeqAfrica collaborators with bioinformatics expertise who work with capacity building projects focused on genomics in LMICs (Survey 1, Annex 1). It was also shared on the SeqAfrica bioinformatics community of practice mailing list, which has a membership of 45. It was also shared on LinkedIn.

Following review of Survey 1 results, we sent an email with an open-ended questionnaire to two large networks working in AMR within the European Union, the European Antimicrobial Resistance Genes – Reference Laboratory Capacity project (EURGen-RefLabCap) network and the national reference laboratories of the European Union Reference Laboratory for Antimicrobial Resistance in Food, Feed, and Animals network (Survey 2, Annex 2) in order to obtain a broader understanding of the main tools that bioinformaticians use. These networks, which are coordinated by our research group, were chosen because they consist of quality-controlled laboratories with strong bioinformatics experience. While these experts work in high-income countries, this survey was intended merely to identify which tools were in the bioinformatics landscape, with the intention of thereafter evaluating if any of the tools mentioned were applicable to the LMIC target context. For survey 2, 80 individuals were contacted through a group email, requesting information about the pipelines that they use in their work.

The responses to the two surveys were analyzed in R (v4.4.3) using the tidyverse package. Tool names were standardized to ensure consistency across entries, and reported categories were mapped to predefined analytical capability classes (AMR, assembly, QC, phylogenetics, base-calling, typing, metagenomics and viral analysis). Interface modality was classified as command-line, GUI, combined command-line and GUI, or other, based on the reported interface description. Unique tools were then enumerated within each capability-modality combination to generate summary statistics of both absolute counts and relative proportions. Where multiple tools were reported within a single entry, these were separated into individual records prior to analysis. Descriptive summaries were additionally generated to quantify tool mentions, interface distributions, and category frequencies.

After reviewing the results of the two abovementioned surveys, we held an expert elicitation workshop to further investigate the promising tools identified in the surveys and to ask experts and users to discuss the applicability of these tools to their work providing bioinformatics capacity strengthening in LMICs. The experts invited were diverse with respect to geography, gender, ethnicity and consisted of those who had responded to one of the two surveys or who were members of the SeqAfrica consortium. An AI tool was used to take notes during the workshop. These notes were thereafter reviewed by two of the authors who both attended the workshop.

Finally, the identified tools were evaluated using the above-mentioned criteria in a simple multi-criteria decision analysis (MCDA) framework. The MCDA was defined based on the requirements for AMR surveillance in low-resource settings. Each criterion was scored on a five-point scale, where 1 indicated very poor performance and 5 indicated excellent performance. Criteria were weighted to reflect their relative importance, with higher weights assigned to cost, ease of use, and AMR relevance (weight = 2), intermediate weights assigned to resource requirements and accessibility (weight = 1.5), and a lower weight assigned to interoperability (weight = 1). Tools were first screened using one knock-out criterion. Tools that were not GUI-based were excluded from further consideration.

## 3 Results

**Figure 1:**
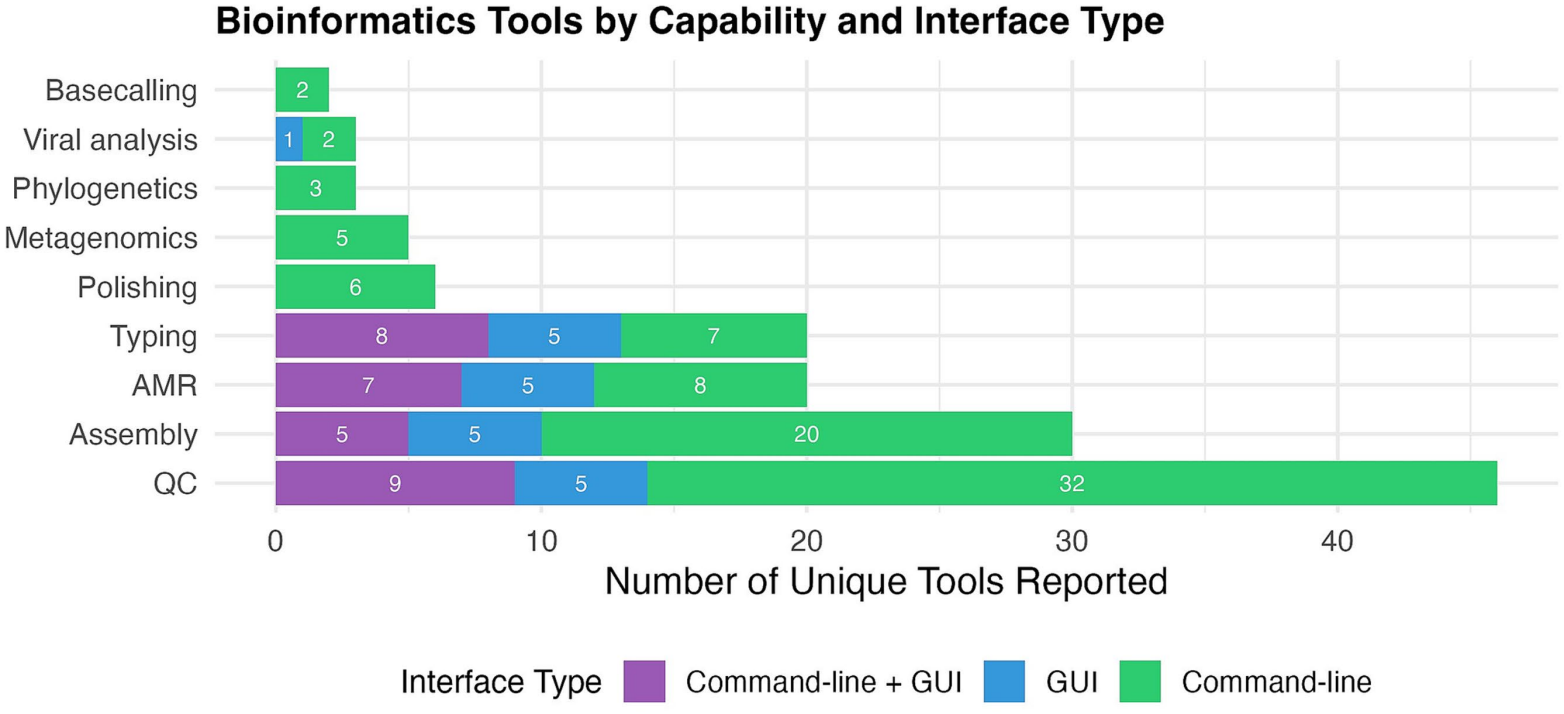
Visualization of unique bioinformatics tools reported in Surveys 1 and 2, categorized by analytical capability and interface type. Each tool is counted only once, regardless of how many times it was mentioned across the surveys, and the figure does not represent frequency of use. Capabilities include antimicrobial resistance (AMR) detection, genome assembly and polishing, quality control (QC), phylogenetics, basecalling, typing, metagenomics, and viral analysis. Interface types were defined as command-line (CLI), graphical user interface (GUI—including both web-based and desktop applications), or tools supporting both CLI and GUI. Tools used across multiple capabilities or interface types were counted once per unique combination. This figure reflects the diversity of tools used across functional categories, rather than their frequency of use.

Across the two surveys, respondents identified 82 unique bioinformatics tools spanning a wide range of functions, including AMR detection (*n* = 25, 15%), assembly (*n* = 35, 20%), QC (*n* = 61, 35%), phylogenetics (*n* = 4, 3%), base-calling (*n* = 2, 1%), typing and annotation (*n* = 24, 14%), metagenomics (*n* = 7, 4%) and viral analysis (*n* = 4, 2%). Of the tools assessed, the majority were command-line based (*n* = 58, 70.7%), followed by tools combining command-line and GUI (*n* = 17, 20.7%). A smaller proportion were GUI only tools (6, 7.3%), while one tool was classified as other (1/82, 1.2%). A summary of the distribution of tools mentioned across both surveys is presented in Figure 1. Survey 1 received 23 responses from bioinformaticians working in seven countries (Ghana, Australia, Denmark, South Africa, USA, Tanzania, Pakistan), with several participants reporting the use of in-house pipelines or combinations of open-source software. Survey two had 77 respondents from individuals working in bioinformatics from across Central, Southern, and Eastern Europe (of 80 individuals contacted) and included a broader set of tools, many of which were command-line based, posing potential barriers for less experienced users or those working in resource limited settings.

**Figure 2:**
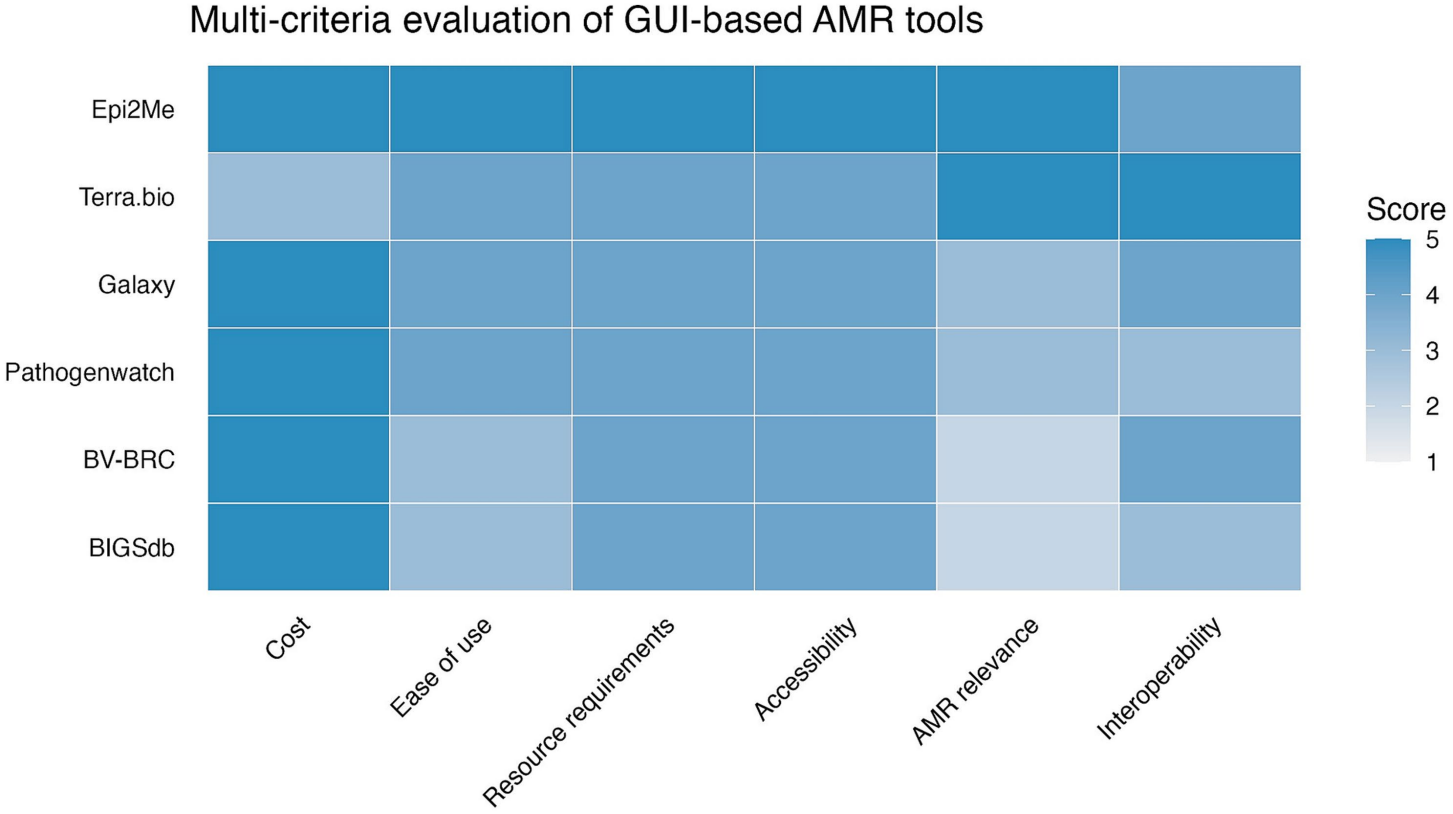
Multi-criteria evaluation of GUI-based AMR tools. Each criterion was scored on a five-point scale, where 1 indicated very poor performance and 5 indicated excellent performance. Criteria were weighted to reflect their relative importance, with higher weights assigned to cost, ease of use, and AMR relevance (weight = 2), intermediate weights assigned to resource requirements and accessibility (weight = 1.5), and a lower weight assigned to interoperability (weight = 1).

All GUI-based tools identified in the surveys (*n* = 6) were evaluated using the multi-criteria decision analysis (MCDA) framework. The tools were evaluated based on desk research and the research team’s trial use of the tools for this review. Among these, EPI2ME achieved the highest overall score, followed by Terra.bio. Galaxy, Pathogenwatch, BV-BRC, and BIGSdb demonstrated strengths in specific domains but scored lower overall against the predefined criteria (Figure 2). While Galaxy, BV-BRC and BIGSdb are GUI-based and widely used, the tools reliance on more complex infrastructure and configuration resulted in lower scores for resource requirements and accessibility within the MCDA framework. Thus, EPI2ME and Terra.bio were prioritized for further investigation based on their overall performance in the MCDA, particularly with respect to cost, accessibility, resource requirements, and AMR relevance. EPI2ME is freely available and supports local execution, while Terra.bio provides low-cost, cloud-based analysis with strong interoperability. Both platforms offer user friendly graphical interfaces and integrated workflows, making them suitable for non-bioinformaticians such as laboratory technicians or clinicians in the target setting.

The virtual workshop was held on Zoom on 15 October 2024 with approximately 20 attendees from across Africa, North America, and Europe. The event began with a presentation by a representative from Theiagen about the Theiagen workflows and their use within the Terra platform. The Theiagen representative shared that the TheiaProk workflows cater to bacterial genome analysis, including *de novo* assembly and taxa-specific analyses (6). The workflows accommodate ONT and Illumina data and can be accessed through command-line interfaces or Terra.bio^1^ a cloud-based bioinformatics platform. Terra was showcased as a user-friendly platform featuring point-and-click workflow execution and scalable cloud resources. The average cost of Terra use is $1/sample for analysis. Terra allows for different types of analyses, integrating tools like Gambit for taxonomic predictions and customizable workflows that are particularly valuable for public health labs worldwide.

The event then continued with a bioinformatics specialist from ONT introducing EPI2ME, a user-friendly platform for real-time genomic analysis (7). The ONT representative stressed the platform’s adaptability and potential for customization to meet diverse laboratory requirements. It is easy to click and install EPI2ME, and workflows can be run on a laptop locally with sufficient RAM and CPUs or remotely on the cloud. EPI2ME can be installed on the same laptop/computer that is running sequencing with a MinION, allowing the user to immediately run a workflow on the sequencing data that is being generated. EPI2ME is also integrated with the ONT GridION and PromethION sequencers. The representative also shared that the platform integrates third-party tools for QC, bacterial genome analysis, and metagenomics, and supports customization for specific user needs, such as AMR gene surveillance or species-specific analyses.

Following the presentations, the group discussed questions including:

- Technical Issues and Tool Performance: Participants discussed issues related to Dorado and concatemer handling, real-time data output in MinKnow.
- Plasmid Detection and Assembly: ONT workflows handle plasmid assembly through reference-based and exploratory *de novo* approaches. The ONT representative highlighted that high coverage and well-prepared libraries are essential for capturing small plasmids and AMR gene variants accurately.
- Species-Specific Concerns: ONT’s performance for low guanine-cytosine (GC)-content species (e.g., *Campylobacter*) was noted to be robust, leveraging tools with enhanced Q-scores (Q55, Q60). Multiplexing guidelines and barcoding protocols were emphasized to maintain data quality across diverse sample types.
- Barcode and Read Issues: The ONT representative explained that due to the single-strand nature of Nanopore sequencing, it is common to have reads with only one barcode, and Dorado can still detect these effectively. Wet-lab factors such as adaptor and barcode ligation efficiency can influence this. Following protocols carefully - sometimes with extended incubation - can improve results. Additionally, the representative confirmed that Dorado has supported splitting reads with middle adapters for amplicon sequencing since May 2024.

Through the discussion, participants stressed the need for modular training programs that cater to non-bioinformaticians, especially in frontline diagnostics. Terra and EPI2ME’s intuitive interfaces were praised for facilitating adoption in resource-limited settings. Discussions also addressed the difficulty and importance of sustaining genomic tools, with a focus on updating algorithms, databases, and workflows. During the discussion, questions arose about the relative performance of tools for AMR determinant detection like ResFinder and AMRFinderPlus. Participants discussed the importance of benchmarking workflows and ensuring bioinformatic tools are vetted for reliability and accuracy. The group also discussed limitations of analytical tools in LMICs. For example, while platforms like Terra and EPI2ME enable cloud-based collaboration and storage, bandwidth constraints in low-resource settings necessitate complementary local solutions.

Using the input from the two surveys and expert elicitation, the following conclusions, as described in Table 1 can be made about each of the most common tools.

**Table 1:** A comparison of the Terra.bio and EPI2ME pipelines.

Criteria	Terra.bio features	EPI2ME features
Cost	$1/sample	Free
Ease of use: *Is the tool intuitive/easy-to-use?*	Easy to intermediate: Interface is slightly more complex with a wide variety of options.	Easy: User interface is click and play. Users must only select workflow and parameters.
Resource requirements: *Can the tool run on a standard computer?*	N/A- runs on the cloud.	Can run locally on laptop or in the cloud. Generally requires a Windows 10 or higher; 8 CPU and 32 GB RAM, but requirements vary based upon the workflow being run locally.
Accessibility: *Can the tool operate offline or on a local server?*	Requires either an internet connection or a server.	Can operate offline.
Interoperability: *Can the tool be used with other systems/workflows?*	Yes. Terra supports open-source workflows written in WDL (Workflow Description Language), which can be easily imported, customized, and run within the Terra platform. Users can integrate their own workflows or use publicly available ones.	Yes. All workflows are open-source. EPI2ME supports custom workflows written in Nextflow. As long as a new workflow is written in Nextflow and follows the required structure, it can be integrated into EPI2ME.

## 4 Discussion

This review was motivated by the urgent need to address genomic AMR surveillance in LMICs, where centralized sequencing infrastructure is often too slow for real time outbreak detection (3, 8, 9). The process described in this paper was designed to identify a user-friendly analytical platform for ONT data. The findings from two surveys and an expert elicitation workshop revealed a fragmented landscape of many analytical tools currently in use, often with overlapping purposes (9, 10). While many respondents relied on in-house and/or command-line pipelines, tools such as Terra.bio and EPI2ME emerged as promising candidates for broader adoption due to their user-friendly GUI interfaces and potential for integration into existing workflows.

Our comparison demonstrates that EPI2ME excels in accessibility and ease of use, offering a free, offline-capable platform with a simple click-and-play interface suitable for resource-limited settings. Its offer of local deployment on the same device used for ONT sequencing makes it particularly attractive in bandwidth-constrained settings. ONT FASTQ files typically range from ~50 MB to several GB per sample, meaning upload to cloud platforms such as Terra may require several hours on standard or low-bandwidth connections and may be impractical in settings with unstable internet access. In contrast, tools such as EPI2ME support local analysis on sequencing laptops, eliminating the need for large data transfers and making them more suitable for low-resource or field-based laboratories.

Importantly, EPI2ME’s foundation in the Nextflow workflow engine provides a significant advantage in terms of reproducibility, modularity, and scalability. A recent review by Langer et al. (10) emphasized that platforms built on Nextflow, especially those aligned with the nf-core framework, are better equipped to meet the FAIR principles (Findability, Accessibility, Interoperability, Reusability) compared to GUI-based platforms such as Galaxy. The growing global adoption of Nextflow and Snakemake pipelines and the plateau in Galaxy use further supports the decision to prioritize a Nextflow-based platform such as EPI2ME for long-term implementation (10). Furthermore, as EPI2ME is developed by ONT, it is expected to be maintained and in line with ONT software releases, ensuring greater long-term pipeline stability.

Also of note in regards to sustainability, several of the workflows underpinning the EPI2ME platform, meaning that they can also be executed via the command line or in alternative computational environments if required. This reduces vendor lock-in risk by allowing users to migrate to non-ONT infrastructure while using the same workflows. In addition, because EPI2ME relies on standard data formats such as FASTQ and assembled genome files, outputs remain fully interoperable with other tools.

Terra.bio stands out for its cloud-based scalability and strong interoperability. However, Terra.bio requires internet access, has a slightly steeper learning curve, and incurs cloud usage costs, which may pose a barrier in resource-limited settings and could also create data security concerns. While both EPI2ME and Terra.bio employ user-centric data ownership models, whereby data remains under the control of the individual that uploads the data, Terra.bio’s use of the Google Cloud Platform does imply greater risk than EPI2ME, which can be operated offline. As AMR genomic data may contain sensitive information, the level of data security offered is an important factor in choosing a tool.

Nonetheless, both tools are actively maintained and support integration with other workflows, making them strong candidates for decentralized AMR genomic surveillance depending on local infrastructure and user expertise (2, 4). Indeed, the PulseNet International project has begun introducing the ONT platform across Asia and Africa and is training users in Terra.bio (11, 12).

The availability of well-maintained, user-friendly platforms not only facilitates decentralized genomic analysis, but also has the potential to accelerate surveillance efforts, inform public health policy, and support integrated One Heath approaches. By enabling timely and accessible data interpretation across human, animal and environmental health sectors, these platforms contribute to more responsive public health systems (13–15). Many existing bioinformatics tools were originally designed for short-read sequencing and may not be optimized for long-read data. ONT users often rely on dedicated assembly tools that can accommodate the unique characteristics of long reads, including their length and higher error rates. Respondents also noted that laboratories being trained on ONT workflows can also be hindered by technical steps like FASTQ file concatenation (merging), particularly on Windows systems where command-line tools are less accessible. ONT often generates multiple FASTQ files per run, requiring users to merge them before analysis, a step that can be non-trivial for those unfamiliar with command-line environments, despite FASTQ files being the primary input for most downstream analysis pipelines. Notably, both EPI2ME and Terra.bio include automated preprocessing steps such as FASTQ concatenation within their workflows, reducing the technical burden on users and supporting downstream analysis.

Although the EPI2ME bacterial-genomes workflow does not currently support plasmid detection or SNP-based phylogenetic analysis, our conclusions reflect a deliberate prioritization of usability and accessibility in resource-limited settings. These limitations can be addressed through a tiered surveillance framework, in which sequencing outputs (e.g., FASTQ files or assembled genomes) are shared with centralized laboratories for advanced analyses, including plasmid characterization and high-resolution phylogenetics. This model enables sentinel sites to participate in genomic surveillance and outbreak detection, while relying on regional or national hubs for more computationally intensive analyses.

A recurring theme in the workshop discussions was the need for standardization and interoperability of data and workflows. The current diversity of tools and workflows limits data comparability and increases the training burden (3, 10). Establishing a consensus around a core set of interoperable tools or a modular pipeline that can be adapted to local needs would enhance the scalability and sustainability of AMR surveillance efforts across the continent. Sustained tool maintenance is a major challenge, particularly given the rapid evolution of ONT technologies, underscoring the importance of sustained technical support (5, 16).

To address these challenges and move toward greater harmonization, several concrete next steps were identified. Based on the elicitation results and user feedback, SeqAfrica adapted and piloted a user-friendly EPI2ME-based pipeline at selected SeqAfrica sentinel sites to ensure accessibility and relevance in diverse settings. In parallel, SeqAfrica held a collaborative hackathon to adapt existing EPI2ME workflows, incorporating input from workshop participants and resolving technical issues that were highlighted. As part of this effort, SeqAfrica has developed a draft protocol for the use of EPI2ME in AMR surveillance workflows, which was subsequently introduced during training sessions at sentinel sites. These activities aim to support capacity building, facilitate regional adoption of standardized workflows, and lay the groundwork for broader implementation of interoperable bioinformatics solutions on the African continent (17).

While this study provides valuable insights, it is not without limitations. We did not benchmark EPI2ME and Terra.com and therefore cannot speak to the relative accuracy of the tools. Future studies could address this gap by undertaking systematic benchmarking to compare the accuracy and performance of these tools. Likewise, this paper focuses on analytical tool selection and does not address broader surveillance determinants such as antibiotic exposure or upstream drivers of AMR. While such considerations are important to AMR surveillance, they fall outside of the limited scope of this manuscript.

The reliance on self-reported data from surveys may not fully capture the operational realities of all laboratories, and the workshop discussions may reflect the perspectives of more experienced users. Nonetheless, the elicitation process has proven to be a powerful tool for gathering diverse perspectives and identifying practical solutions (4). The expert elicitation process provided critical insights into the practical needs and preferences of genomic surveillance practitioners in LMICs. These findings will inform the development of sustainable, scalable, and context-appropriate bioinformatics solutions for AMR monitoring across Africa.

## Data Availability

The original contributions presented in the study are included in the article/Supplementary material, further inquiries can be directed to the corresponding author.

## Footnotes

1
https://terra.bio
