# Supplementary material for: Bridging the bioinformatics gap: tool selection for decentralized AMR genomic surveillance in Africa
Source: Front Public Health. 2026 Apr 9;14:1756324. doi: 10.3389/fpubh.2026.1756324 (PMC13102816; doi:10.3389/fpubh.2026.1756324)
Supplement: Supplementary file 1 [file Supplementary_file_1.docx]

**Annex 1: Survey 1**

- Do you conduct viral and/or microbial sequencing using Oxford Nanopore Technologies sequencing platform?
- What pipeline do you use for analysis of your ONT sequencing data? Please include a link if relevant.
- What type of experimental strategy does your bioinformatic pipeline for analysis of ONT sequencing allow?
- Whole genome sequencing of single isolates (WGS), metagenomics (16S), Shotgun metagenomics (untargeted sequencing), other
- Does your bioinformatic pipeline allow: species identification, cluster analysis, detection of AMR genes, serotyping, Multilocus sequencing typing (MLST)?
- Where do you process your ONT WGS data? In a cloud server, on a desktop computer, through an HPC, I don’t know
- If you answered cloud server, what cloud server do you use for data processing? Please provide a link if relevant.
- What challenges do you face in ONT data analysis?
- Would you be interested in participating or presenting at a  virtual expert elicitation on ONT data processing tools?
- Please share any additional comments here.

**Annex 2: Survey 2**

If you conduct viral and/or microbial sequencing using an Oxford Nanopore Technologies sequencing platform, what pipeline do you use for analysis?

**Annex 3: Excerpt of Coding Framework used for Thematic Grouping**

**
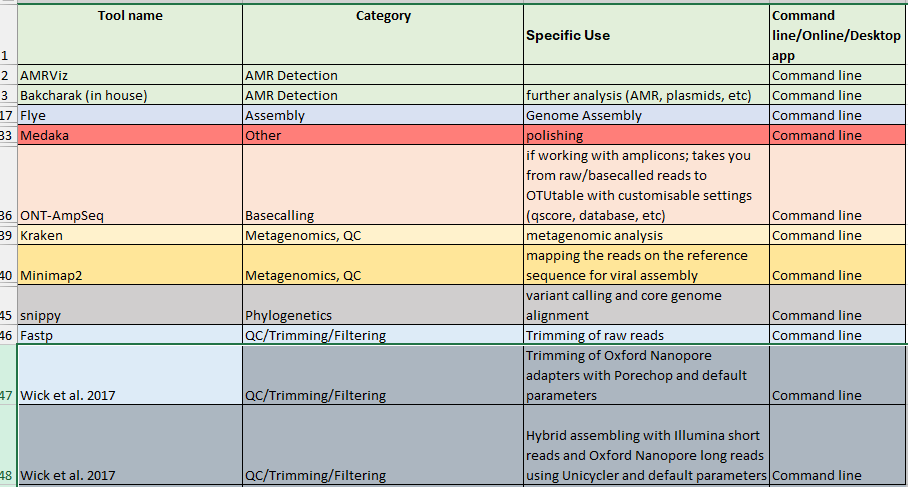
**
